# Interpretation of Discordance Between Non-Hyperemic Pressure Ratios and Fractional Flow Reserve: Potential Mechanisms and Clinical Implications

**DOI:** 10.31083/RCM40417

**Published:** 2025-10-31

**Authors:** Akshay Roy-Chaudhury, Sameer Prasada, George A. Stouffer

**Affiliations:** ^1^Division of Cardiology, University of North Carolina, Chapel Hill, NC 27599, USA; ^2^McAllister Heart Institute, University of North Carolina, Chapel Hill, NC 27599, USA

**Keywords:** fractional flow reserve, coronary artery disease, coronary hemodynamics, resting indices, coronary revascularization

## Abstract

Invasive coronary angiography remains the gold standard for assessing and treating coronary artery disease (CAD). While the decision to intervene on a severely stenotic lesion in acute coronary syndrome (ACS) can be straightforward, assessing the potential benefits of treating an intermediate lesion, especially in patients with stable symptoms, often requires hemodynamic assessment or intravascular imaging. Fractional flow reserve (FFR) is a well-established invasive hemodynamic assessment that is the gold standard for determining the functional significance of intermediate lesions by analyzing the pressure loss across an area of stenosis during maximal hyperemia. The association between the use of FFR and improved clinical outcomes has been validated by numerous clinical trials, leading to societal guidelines for the use of FFR. Recently, invasive hemodynamic indices have been developed that do not require the induction of hyperemia. These non-hyperemic pressure ratios (NHPRs) include the resting full-cycle ratio (RFR), instantaneous wave-free ratio (iFR), diastolic hyperemia-free ratio (DFR), and diastolic pressure ratio (dPR). Clinical studies have suggested “discordance” in FFR and NHPRs in approximately 20% of patients with NHPR-/FFR+ being slightly more prevalent than NHPR+/FFR-. Discordance has been associated with clinical factors, including advanced age, female sex, presence of diabetes, and microvascular dysfunction. Data are inconsistent about whether deferral of revascularization is safe in patients with discordance; however, patients who are NHPR-/FFR+ are more likely to have focal than diffuse disease okand more likely to observe a symptomatic benefit from percutaneous coronary intervention (PCI). Nonetheless, large-scale studies are needed to improve understanding of this discordance, particularly in relation to clinical outcomes.

## 1. Introduction

More than two million percutaneous coronary interventions (PCIs) are performed annually for coronary artery disease (CAD) worldwide [1]. Coronary lesions are visualized by angiography; however, assessing which visually intermediate lesions are causing myocardial ischemia and anginal symptoms can be challenging. Numerous studies have shown that the ability of angiographic assessment to predict the hemodynamic effect of an atherosclerotic lesion is limited, and there is significant inter-operator variability in visual lesion assessment [2, 3]. Physiology-based quantification of lesion severity provides objective data on the hemodynamic significance of stenosis, prognosis, and the effectiveness of PCI in relieving symptoms. Fractional flow reserve (FFR) represents the most widely used invasive hemodynamic tool; however, non-hyperemic pressure ratios (NHPRs) have recently gained popularity. Both of these physiological measurements supplement visual angiographic assessment and assist decision-making during coronary angiography.

## 2. Hemodynamics and FFR Measurement

Myocardial perfusion and coronary blood flow are primarily regulated by the coronary microcirculation, which encompasses the vast majority of the overall coronary vasculature [4]. In a normal physiological state, the resistance provided by the microvasculature is maintained at a level to enable coronary blood flow to meet myocardial metabolic demands. However, when myocardial demand increases, the microcirculation dilates, resulting in decreased resistance, increased blood flow, and enhanced metabolic supply.

The basic principle of FFR is that at minimal resistance, the change in flow is proportional to the change in pressure [5]. FFR is the ratio of distal coronary artery pressure (Pd) to aortic pressure (Pa) during maximal coronary flow and, thus, a surrogate for maximal flow in a diseased coronary artery divided by maximal flow in that artery in the absence of any stenosis.

FFR measurement is performed by advancing a 0.014-inch pressure-sensing coronary wire distal to an angiographically intermediate epicardial coronary lesion of interest. Typically, either an intracoronary adenosine bolus (30–200 µg) or intravenous adenosine infusion (140 µg/kg/min) is administered to minimize microvascular resistance and induce hyperemic coronary blood flow. The pressure wire measures the coronary pressure distal to the lesion, and the guide catheter measures the aortic pressure at the time of maximal hyperemia to calculate FFR ratio, as previously described [5]. By consensus, an FFR value ≤0.80 is considered to indicate a hemodynamically significant epicardial stenosis.

## 3. Clinical Evidence for FFR

Pijls *et al*. [6] compared FFR measurements with non-invasive functional stress tests, as well as quantitative coronary arteriography, in 45 patients with chest pain and moderate coronary stenoses. If the FFR was <0.75, revascularization was performed if the lesion was suitable, after which the FFR was re-measured. For all patients with FFR <0.75, myocardial ischemia was evident on at least one non-invasive test. For those who underwent PCI, the repeat FFR measurements increased to >0.75. In 21 of the 24 patients with a FFR >0.75, all non-invasive tests were negative. The accuracy of FFR in this case was 93%, with positive predictive value (PPV) of 100% and negative predictive value (NPV) of 88% [6].

Numerous studies have assessed clinical outcomes using FFR. In a randomized study of 325 patients referred for PCI, Bech *et al*. [7] compared the deferral or performance of PCI in patients with a FFR >0.75 regarding major adverse cardiac events (MACEs) or freedom from angina for 24 months. No benefit was determined for performing PCI in this population [7]. The DEFER trial was a prospective, randomized trial that included patients with stable chest pain and intermediate coronary stenoses and no evidence of ischemia, following non-invasive testing, who were scheduled to undergo elective PCI. Patients were randomized into either a deferral group or a performance group, with a FFR performed for all patients. The trial resulted in three groups: (1) Patients with a FFR >0.75, where PCI was deferred (deferral); (2) patients with a FFR >0.75, where PCI was performed (performance); (3) patients with a FFR <0.75, where PCI was performed regardless of group randomization [7]. After long-term follow-up at 5 years and 15 years, the deferral of PCI was shown to promote excellent outcomes, with similar mortality rates and a lower rate of myocardial infarction (MI) compared to the PCI performance group. The risk of death or MI from a hemodynamically nonsignificant lesion was observed to be <1% per year; further interventions did not decrease this risk. The greatest risk of cardiac adverse events was associated with the reference group patients who had a FFR positive lesion, even if PCI was performed [8, 9]. Collectively, these trials established the safety of deferring PCI in terms of long-term outcomes for patients with a FFR >0.75.

The FAME (Fractional Flow Reserve versus Angiography for Guiding Percutaneous Coronary Intervention) trial was a landmark study for FFR [10], as this research examined whether routine assessment of hemodynamic significance using FFR would improve outcomes in patients with multivessel coronary disease undergoing PCI. In this multicenter, randomized trial involving 1005 patients across 20 centers in the United States and Europe, patients were randomized to angiography-only-guided PCI or FFR-guided PCI. For the angiography-guided group, patients underwent PCI for all indicated lesions as suggested by visual angiography. In the FFR-guided group, patients underwent PCI only for those lesions where FFR was ≤0.80. The primary endpoint was MACEs at 1 year, which occurred in 91 patients (18.3%) in the angiography-guided group and 67 patients (13.2%) in the FFR-guided group (relative risk (RR) 0.72, 95% confidence interval (CI) (0.54–0.96); *p* = 0.02). Furthermore, FFR-guided PCI decreased stent usage, contrast usage, and cost while achieving similar or improved mobility with no decrease in quality of life. Interestingly, 37% of the lesions classified as “severe” angiographically in the FFR arm were found to be hemodynamically insignificant and were, therefore, treated medically.

FAME 2 compared outcomes in patients with hemodynamically significant coronary lesions randomized to optimal medical therapy versus optimal medical therapy and PCI. However, the study was halted prematurely because the primary composite endpoint of death, MI, or urgent revascularization was significantly higher in the optimal medical therapy alone group [11]. The premature stoppage was criticized as the difference in composite outcome was largely driven by the need for more urgent revascularization in the optimal medical therapy arm.

Importantly, FFR has also demonstrated benefit in the acute MI setting for non-culprit lesions. Physiology-guided complete revascularization of non-culprit lesions has been associated with improved cardiovascular outcomes in patients with ST-elevation myocardial infarction (STEMI) and non-ST-elevation myocardial infarction (NSTEMI) compared to culprit-only revascularization [12, 13, 14].

## 4. Guideline-Supported FFR Use

The use of FFR-guided revascularization in patients with chronic coronary syndromes (CCSs) is supported by a large evidence base, and there is guideline support from multiple societies. The European Society of Cardiology currently issues a Class Ia recommendation for the use of FFR in patients with intermediate-grade stenosis (40–90%) in the absence of evidence of ischemia on non-invasive testing and a Class IIa recommendation for the use of FFR-guided PCI in patients undergoing multivessel PCI [15]. The 2021 AHA/ACC guidelines for coronary artery revascularization similarly recommend a Class Ia use of FFR in guiding decisions on PCI in patients with angina and angiographically intermediate stenosis [16].

FFR is also useful in evaluating non-culprit lesions in patients with acute coronary syndrome (ACS). In patients with STEMI, multiple trials have demonstrated that FFR-guided complete revascularization is associated with improved cardiovascular outcomes, particularly a decreased need for subsequent revascularization [12, 13, 14]. However, the optimal timing of the physiological assessment remains unclear. The 2023 European Society of Cardiology (ESC) ACS guidelines [17] provide a Class III recommendation against the functional evaluation of non-culprit lesions during primary PCI procedures for STEMI patients with multivessel disease. In contrast, the 2025 ACC/AHA guidelines [18] for ACS do not mention the use of hemodynamic assessment of non-culprit arteries during primary PCI in patients with STEMI. However, evidence exists and has been well-documented that FFR is useful in patients presenting with NSTEMI [19]. Both the 2023 ESC ACS guidelines and the 2025 ACC/AHA guidelines for ACS recommend a Class IIB approach for physiology-based revascularization of non-culprit lesions in patients with NSTEMI and multivessel disease [17, 18].

## 5. Non-Hyperemic Pressure Ratios

While there is strong evidence for employing FFR as a viable tool for hemodynamic assessment of coronary ischemia, this method requires the administration of vasodilators, which adds time, cost, and further risk. Furthermore, FFR requires the assumption that microvascular resistance is minimized during hyperemia to ensure that pressure and flow through a stenotic lesion are proportional and, thus, that a decrease in pressure is equivalent to a reduction in flow. However, while FFR measurements are averaged over multiple cardiac cycles, fluctuations in coronary resistance still occur between systole and diastole [20]. As such, identifying a period during the cardiac cycle when coronary resistance is constant and minimal would negate the need for inducing hyperemia. This led to the development of the various NHPRs (Table 1). All the NHPRs compare the pressure in the distal portion of the coronary artery to the pressure in the aorta in the basal state, but vary depending on which part of the cardiac cycle is sampled. Moreover, these NHPRs can be divided into whole-cycle (resting Pd/Pa and resting full-cycle ratio (RFR)) versus phase-specific ratios (instantaneous wave-free ratio (iFR), diastolic hyperemia-free ratio (DFR), and diastolic pressure ratio (dPR)).

**Table 1.** **Non-hyperemic pressure ratios**.

Index	Definition	Cut-off for ischemia
Resting Pd/Pa	Lowest mean Pd/Pa over entire cardiac cycle	0.91 or 0.92
iFR: instantaneous wave-free ratio	Pd/Pa during the wave free period of diastole	0.89 or 0.90
dPr: diastolic pressure ratio	Pd/Pa during diastole	0.89
DFR: diastolic hyperemia-free ratio	Average Pa/Pd during the period when Pa < mean Pa	0.89
RFR: resting full-cycle ratio	Lowest instantaneous Pd/Pa over entire cardiac cycle	0.89

Pd, distal coronary artery pressure; Pa, aortic pressure.

Compared to the hyperemic indices (e.g., FFR), the pressure gradients in the NHPRs are smaller, which makes these gradients more susceptible to errors due to pressure drift, hydrostatic effects, hemodynamic changes, and electronic noise.

The iFR is the best-studied NHPR, as this ratio utilizes a wave-free period during diastole, which is associated with minimal coronary resistance. Moreover, iFR was found to be reproducible and accurate compared to FFR for identifying hemodynamically significant stenotic lesions. Both the DEFINE-FLAIR and iFR-SWEDEHEART trials demonstrated that an iFR-guided strategy was non-inferior to FFR guidance with respect to major adverse cardiovascular events at 1 year and 5 years in patients with intermediate coronary lesions referred for PCI. Importantly, in both trials, the use of iFR was associated with a greater deferral of revascularization compared with the use of FFR for physiological guidance [21, 22]. Several other NHPRs have since been studied, with results showing a high correlation between ratios and no measurable difference between modalities [23, 24, 25].

## 6. Interpretation of Discordance

In many patients undergoing a physiological evaluation of an intermediate coronary lesion, both NHPR and FFR are measured. Concordance is present when both physiological indices are correlated—either both NHPR and FFR are consistent with a non-hemodynamically significant lesion or both NHPR and FFR are consistent with a hemodynamically significant stenosis. In the former group of patients, a consensus was found that to defer revascularization is safe. In contrast, revascularization was generally recommended in the latter group. Discordance is present when results of NHPR and FFR do not agree; depending on the population studied, discordance existed between FFR and NHPRs in approximately 20% of patients with a range of 11–28% (Table 2, Ref. [26, 27, 28, 29, 30, 31, 32, 33, 34, 35, 36, 37, 38, 39, 40]) [25, 26, 27, 28, 29, 41]. The benefits of revascularization in these patients are less well understood.

**Table 2.** **Studies of discordance between FFR and NHPRs**.

Study	Number and type of patients enrolled	Demographics	Concordance and discordance	Comparison of Groups 2 and 3
Lee JM *et al*. [33]	975 arteries in 393 patients	Mean age = 64 ± 10 years; males = 77% and DM = 36%	Group 1 = 74% Group 2 = 3% Group 3 = 8% Group 4 = 14%	Females and DM were higher, the reference vessel diameter was smaller, and stenosis was more severe in Group 2 compared to Group 3.
Lee SH *et al*. [39]	840 arteries in 596 patients	Mean age = 62 ± 10 years; males = 72% and DM = 28%	Group 1 = 71% Group 2 = 5% Group 3 = 8% Group 4 = 16%	For CFR, RRR, and IMR, Group 2 was similar to Group 4, and Group 3 was similar to Group 1.
Goto R *et al*. [34]	220 intermediate coronary lesions in 156 patients	Mean age = 73 years; males = 82% and DM = 40%	Group 1 = 52% Group 2 = 8% Group 3 = 11% Group 4 = 29%	Left main or LAD lesion, hemodialysis, and peripheral artery disease were associated with Group 2 and higher hemoglobin levels, and the absence of DM was associated with Group 3 in multivariable regression analysis.
Kato Y *et al*. [40]	573 intermediate coronary lesions in 410 patients	Mean age = 70 ± 11 years; males = 76% and DM = 44%	Group 1 = 37% Group 2 = 7% Group 3 = 21% Group 4 = 36%	Female, LAD lesion, and hemodialysis were significant predictors of Group 2, and body surface area and non-LAD lesions were significantly associated with Group 3 in multivariate analyses.
Legutko J *et al*. [38]	157 arteries in 101 patients	Median age of 66 years; 74% male and DM = 40%	Group 1 = 46% Group 2 = 4% Group 3 = 24% Group 4 = 26%	CMD was higher, and CFR was lower in discordant arteries. Main predictors of RFR/FFR discordance were higher age, presence of CMD, lower CFR, and lower RRR values in univariate analysis.
Wienemann H *et al*. [26]	712 coronary lesions in 617 patients	Mean age = 69 years; 73% male and 27% DM	Group 1 = 56% Group 2 = 9% Group 3 = 13% Group 4 = 22%	Predictors for being in Group 3 included a prior PCI in the target vessel, diameter stenosis, and non-LCX lesions.
Warisawa T *et al*. [29]	International multicenter registry of 360 lesions in 345 patients	Mean age = 64.4 ± 10.3 years; males = 76% and DM = 31%	Group 1 = 35% Group 2 = 11% Group 3 = 11% Group 4 = 43%	A predominant focal disease pattern was associated with Group 3, and a predominant diffuse disease pattern was significantly associated with Group 2. The pattern of disease was the only factor influencing FFR/iFR discordance.
Kovarnik T *et al*. [28]	International multicenter registry of 1884 lesions from 1564 patients	Groups 1 and 4: mean age = 69.0 ± 9.7 years; male = 70.3% and DM = 39%	Group 1 = 41% Group 2 = 7% Group 3 = 14% Group 4 = 39%	Male, younger age, and right coronary artery (RCA) lesion were predictors for Group 3, and renal insufficiency, hemoglobin level, and smoking were predictors for Group 2.
Dérimay F *et al*. [31]	587 patients from the CONTRAST study	In the four groups, mean age varied from 63 to 68 years; male proportion from 67 to 78% and DM from 23 to 29%	Group 1 = 42% Group 2 = 9% Group 3 = 12% Group 4 = 38%	A multivariate analysis indicated that left main or proximal LAD lesion, more severe stenosis, younger age, and slower heart rate were predictors in Group 3, and absence of a beta-blocker, older age, and less severe stenosis were predictors in Group 2.
Faria *et al*. [32]	Post hoc analysis of 690 lesions from 591 patients in the ADVISE II trial	Mean age = 64 ± 11 years; male = 69% and DM = 35%	Group 1 = 56% Group 2 = 10% Group 3 = 8% Group 4 = 26%	Hyperemic responses were age dependent, FFR values increased with patient age, while iFR values remained constant. There was an association between younger age and being in Group 3.
Scoccia A *et al*. [30]	1092 arteries in 813 patients from a single center	Median age was 65 (IQR: 59–73) years; 70% were male and DM = 26%.	Group 2 = 10% Group 3 = 12%	There was a higher prevalence of LAD lesions, and mean values of FFR and dPR were significantly lower in discordant vs. concordant arteries. The difference between dPR and the cut-off value of 0.89 was the only independent predictor of discordance on multivariable analysis.
Cook CM *et al*. [27]	366 disease arteries from 291 patients and 201 unobstructed arteries from 153 patients as part of the IDEAL study	Mean age = 61 ± 10 years; male = 69% and DM = 22%	Group 1 = 55% Group 2 = 8% Group 3 = 6% Group 4 = 30%	Discordance was explained by differences in hyperemic coronary flow velocity. Patients in Group 3 had similar coronary flow characteristics to those with angiographically unobstructed vessels.
Stegehuis V *et al*. [35]	647 intermediate lesions in 593 patients from the IDEAL registry, and the DEFINE-FLOW study	Mean age was 65 ± 10 years; 77% male and DM = 26%	Group 1 = 56% Group 2 = 6% Group 3 = 9% Group 4 = 29%	Patients in Group 3 were younger and more frequently active smokers, while the prevalence of DM was higher in Group 2 (*p* = 0.08).
Yamazaki T *et al*. [36]	408 intermediate lesions in 277 patients	Mean age = 72 ± 10 years; males = 83% and DM = 44%	Group 1 = 45% Group 2 = 11% Group 3 = 11% Group 4 = 33%	Group 2 had higher rates of diabetes, hemodialysis, CKD, and severe aortic stenosis than Group 3. Log-transformed BNP was significantly higher, while hemoglobin and eGFR were significantly lower in Group 2 than in Group 3.
Pisters R *et al*. [37]	515 intermediate lesions in 356 patients	Mean age = 67 ± 10 years; males = 69% and DM = 21%	Group 1 = 62% Group 2 = 16% Group 3 = 3% Group 4 = 19%	Differences in demographics, comorbidities or anatomy between groups were not reported.

All studies were single-center unless noted. Group 1 = FFR >0.80 and normal NHPRs; Group 2 = FFR >0.80 and abnormal NHPRs; Group 3 = FFR ≤0.80 and normal NHPRs; Group 4 = FFR ≤0.80 and abnormal NHPRs. BNP, B-type natriuretic peptide; DM, diabetes mellitus; CFR, coronary flow reserve; CKD, chronic kidney disease; FFR, fractional flow reserve; dPR, diastolic pressure ratio; iFR, instantaneous wave free ratio; PCI, percutaneous coronary intervention; RFR, resting full-cycle ratio; LAD, left anterior descending; CMD, coronary microvascular dysfunction; IMR, index of microcirculatory resistance; IDEAL, Iberian–Dutch–English study.

Discordance occurs whenever either the NHPR is normal and FFR ≤0.80 or the NHPR is abnormal and FFR >0.80. Notably, no consensus currently exists on how to label patients with different types of discordance; however, for this paper, we adopted the convention that patients can be divided into four groups depending on the values of the NHPRs and FFR: Group 1 = FFR >0.80 and normal NHPRs; Group 2 = FFR >0.80 and abnormal NHPRs; Group 3 = FFR ≤0.80 and normal NHPRs; Group 4 = FFR ≤0.80 and abnormal NHPRs. In general, the size of Groups 2 and 3 are similar, with Group 3 being slightly larger than Group 2.

There are several potential explanations, most likely overlapping, for why some patients have discordant results. First, the cut-off values for FFR and NHPRs are arbitrary; thus, that most discordance occurs near these cut-off values is unsurprising. The PREDICT trial retrospectively measured dPR in 813 patients who underwent FFR measurement of intermediate coronary stenoses using dedicated software, of whom two-thirds presented with ACS and one-third with stable angina [30]. A total of 22% of the lesions had discordant findings between FFR and dPR, with the strongest predictors for discordance being a dPR value near the cut-off (0.89) and the lesion being in the left anterior descending (LAD) coronary artery. In a retrospective analysis of almost 500 patients, Mamas *et al*. [42] found that PPV and NPV for the NHPRs predicting a positive or negative FFR increased to >95% when only resting pressure ratios of <0.88 and >0.95 were considered.

Several clinical factors are also associated with discordant results, including age, gender, and diabetes mellitus (DM). A sub-study of the FAME trial found that FFR was significantly higher in older patients, and the proportion of functionally significant lesions was significantly lower at a given stenosis severity compared to younger patients. The effect of age on discordance between the NHPRs and FFR was demonstrated in a study by Dérimay *et al*. [31], who found that younger patients were significantly more likely to be in Group 3. In comparison, older patients were more likely to be in Group 2. Similar results were presented by Faria *et al*. [32], who found a decrease in the proportion of patients with an abnormal FFR despite a normal iFR as age increased.

Several studies have found that gender and DM are associated with discordance and, in particular, that females and patients with DM have a higher representation in Group 2. In the FiGARO trial, females comprised 37% of patients in Group 2, compared to 15% in Group 3. Meanwhile, sex, age, and lesion location in the right coronary artery were identified as predictors of discordance in the multivariable logistic regression analysis. Similarly, females comprised 37% of Group 2 and 6% of Group 3 in a sub-study of the 3V FFR-FRIENDS study. In multivariable generalized estimating equation modeling, female, DM, smaller reference vessel diameter, and greater percent diameter stenosis were significantly associated with Group 2, and males, absence of DM, and lower percent diameter stenosis were significantly associated with Group 3 [43]. Higher rates of DM in Group 2 compared to Group 3 represent a consistent finding across numerous studies [27, 33, 34, 35, 36]. Various other comorbidities have been associated with discordance, including hemodialysis and peripheral artery disease [34], CKD, and severe aortic stenosis [36] and active smoking [35].

Differences in coronary flow reserve (CFR) and coronary microvascular dysfunction (CMD) have been linked to discordance in several studies. In a study of 101 patients with stable angina and intermediate coronary stenoses in which 28% of lesions had discordant NHPR/FFR values, rates of CMD were higher (64% vs. 41%; *p* = 0.01) and CFR was lower (median 1.95 (interquartile range (IQR): 1.37, 2.30) vs. 2.10 (IQR: 1.50, 3.00); *p = 0.*030) in discordant lesions compared to concordant lesions. The two strongest predictors for discordance included higher age and presence of CMD [38]. Evidence that hyperemic responses and/or CFR are greater in Group 3 than in Group 2 was provided by Lee SH *et al*. [39], Petraco *et al*. [44], and Cook *et al*. [27]. In particular, Cook *et al*. [27] found that CFR and hyperemic flow velocity in Group 3 were similar to those in Group 1 and to unobstructed coronary arteries. Conversely, Groups 2 and 4 had similar CFRs. Age and microvascular dysfunction have been suggested as mechanisms for blunted hyperemic responses. Faria *et al*. [32] found that hyperemic response to adenosine is age dependent, with hyperemic flow decreasing with age (and, thus, FFR values increasing with age), while iFR values remained constant across the age spectrum [32]. Finally, while not directly evaluating patients with discordant NHPR/FFR results, Ahn *et al*. [45] reported that a preserved FFR and low CFR were associated with increased microvascular resistance, while patients with a low FFR and preserved CFR had modest epicardial stenosis and preserved microvascular function.

In the PREDICT trial [30], 22% of stenoses had discordant findings between FFR and dPR. Clinical factors that are associated with CMD (female sex, chronic kidney disease, peripheral artery disease, abnormal ejection fraction (EF)) were associated with Group 2. As noted above, other studies have corroborated that factors associated with CMD, such as female sex, end-stage renal disease requiring dialysis, peripheral artery disease, DM, and tobacco use, are associated with the FFR-/NHPR+ subtype [27, 28, 34, 40, 46]

There is emerging evidence that the anatomic pattern of CAD (diffuse vs. focal) predicts the discordance subtype. The REVEAL-iFR study enrolled 355 patients with CCS and intermediate lesions who had FFR and iFR measured in addition to pull-back pressure gradient index as estimated by an angiography-based virtual pressure pull-back curve, which can categorize lesions as diffuse or focal stenoses [47]. Patients in the FFR+/iFR- subtype (Group 3) had a predominantly focal disease pattern (76%), while those in the FFR-/iFR+ subtype (Group 2) almost always had a diffuse disease pattern (96%). This association is presumably due to different flow dynamics in these settings. These findings are consistent with data from two prior registries, the Multicenter AJIP and Verona University Hospital, which used both angiography-based and wire-based pressure pull-back curves, and found that Groups 2 and 3 had a majority of diffuse and focal disease patterns, respectively [29, 48]. The association is particularly strong for Group 2 in each of these studies. Previous work has suggested that PCI is more likely to offer clinical benefit for focal lesions; therefore, the subtype of FFR+/iFR- lesions may be better suited for intervention [47, 49, 50, 51, 52].

Lastly, some studies have found an association between discordance and the location of the atherosclerotic disease within an epicardial coronary artery and/or the specific coronary artery involved. The VERIFY 2 study, which included 197 patients with 257 moderate coronary stenoses, found iFR/FFR discordance in 28% of lesions in the proximal portion of the artery, compared to 15% of lesions that were more distal [53]. In contrast, the CONTRAST study of 763 patients reported that left main and proximal left anterior descending artery lesion locations, compared with other lesion locations, were associated with discordance between NHPR (iFR or Pd/Pa) and FFR [54]. Several other studies have found an association between discordance and disease in the LAD [34, 40, 54] or right coronary artery (RCA) [28].

## 7. Discordance in STEMI Patients

Half of the patients presenting with STEMI are found to have multivessel disease and non-culprit stenotic lesions. A sub-study of the REDUCE-MVI (Reducing Micro Vascular Dysfunction in Revascularized STEMI Patients by Off-target Properties of Ticagrelor) trial, involving 73 patients with STEMI and multivessel disease [55], found that non-culprit FFR values were higher at the time of STEMI presentation than when measured one month later. Blunted hyperemic responses were more common in patients with larger infarct size, as well as lower left ventricular ejection fraction, and more microvascular injury (concomitant with suppressed CFR in the acute setting). Several factors contribute to a blunted hyperemic response, including increased left ventricular end-diastolic pressure, augmented neurohormonal activation, myocardial edema, and decreased adenosine receptor sensitivity in the acute setting [56].

## 8. Clinical Outcomes

There is limited data on prognostication for individuals who have discordant values between FFR and NPHR. A sub-study of the 3v FFR-Friends study included 821 intermediate lesions in 374 patients, for which no revascularization was performed, and for which both FFR and iFR were measured. The two-year MACE rates were 2.4% in Group 1 (n = 706), 3.3% in Group 2 (n = 32), 2.5% in Group 3 (n = 40), and 11.6% in Group 4 (n = 43). Only Group 4, which presented concordant abnormal results, showed a significantly higher risk of MACEs [25].

A subsequent sub-study of 1024 vessels from 435 patients in the 3v FFR-Friends study found that the risk of a vessel-oriented composite outcome (VOCO) at a follow-up of 5 years was higher in 57 patients with discordant results who did not have revascularization than in the 688 patients with concordant negative results and the event rate was equivalent to that of the 127 patients with concordant positive results who underwent revascularization [57]. The higher event rate was primarily driven by vessel-related ischemia-driven revascularization in lesions with positive NHPRs or FFR. Similar findings were observed in a Korean study of 596 patients with deferred intermediate coronary lesions who underwent measurements for FFR and iFR [39]. Patients with discordant FFR and iFR indices did not have significantly worse patient-oriented composite outcomes at 5 years compared to patients with concordantly normal indices. Both of these trials were limited by a relatively low number of patients with discordance and low event rates, and, thus, further investigations are needed.

In contrast to the results of these studies, a meta-analysis of six trials that included 9854 intermediate lesions with determination of both FFR and NHPR (two studies with 1563 lesions used iFR; two studies with 965 lesions used RFR; one study with 4899 lesions used Pd/Pa; one study of 2427 lesions used a mixture of NHPRs), found that deferral of PCI was associated with an increased risk of death or MI in both Groups 2 and 3 compared to Group 1 [58]. In an exploratory analysis, PCI reduced the primary endpoint in Group 3 but not in Group 2.

Several studies have examined outcomes in patients with CFR and FFR discordance. A study of 157 intermediate coronary stenoses in 157 patients who did not undergo revascularization found that discordant results between FFR and CFR occurred in 37% of lesions and were associated with differences in microvascular resistance during basal and hyperemic conditions [59]. Over 10 years of follow-up, a normal FFR with an abnormal CFR was associated with significantly increased MACEs, while an abnormal FFR with a normal CFR was associated with equivalent clinical outcomes compared with concordant normal results of FFR and CFR. Similar results were obtained in a study of 220 stenoses in 220 patients, all of whom had a FFR ≤0.80 and underwent PCI [60]. At a median follow-up of 24.3 months, MACE rates were higher in patients with lower pre-PCI CFR, and stepwise multivariable Cox regression analysis showed that low pre-PCI was an independent predictor of adverse events during follow-up.

## 9. Conclusion

**Fig. 1.**
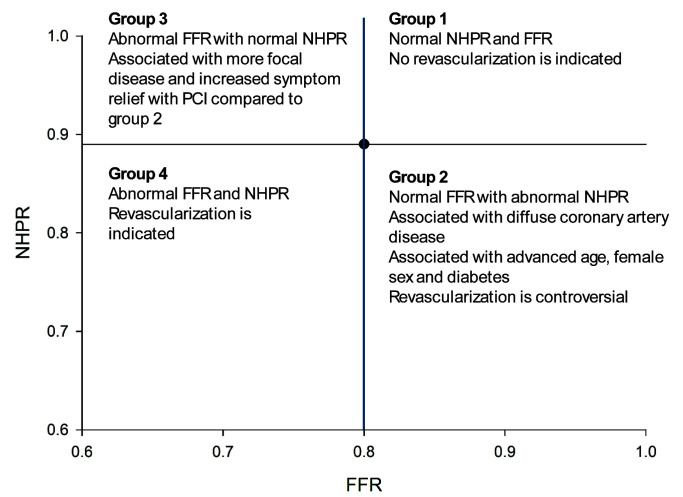
**Schematic of the four quadrants that patients can be in with measurement of both non-hyperemic pressure ratios (NHPRs) and fractional flow reserve (FFR)**.

Widespread availability of coronary angiography and the development of advanced interventional techniques have resulted in significant improvement in outcomes for patients with acute coronary syndrome and CCS. The need for a more detailed assessment to determine the clinical significance of intermediate stenotic lesions led to the development of hemodynamic invasive evaluations, including hyperemic and non-hyperemic indices. While both techniques are commonly utilized, sometimes concurrently, a discordance rate of approximately 20% exists between the two indices, creating uncertainty regarding the benefits of revascularization (Fig. 1). In this review, we have subtyped discordant groups, categorizing FFR-/NHPR+ as Group 2 and FFR+/NHPR- as Group 3. Several mechanisms have been identified that may contribute to discordance, including clinical factors of advanced age, female sex, and the presence of diabetes or chronic kidney disease (CKD). These same factors predispose patients to CMD, which is a major driving force for Group 2 physiology. Coronary factors, such as anatomic patterns of disease, also contribute, as focal stenoses are more often seen in Group 3 and diffuse disease in Group 2. There are limited data on outcomes for patients with discordant values, which preclude definitive recommendations. Preliminary data from smaller studies have suggested no difference in MACEs with revascularization versus deferral in Groups 2 or 3. However, Collet *et al*. [52] reported that revascularization resulted in greater improvements in FFR, Pd/Pa, CFR, and anginal symptoms in patients with focal disease compared to those with diffuse disease, suggesting a differential benefit in terms of anginal relief with revascularization in Group 3 versus Group 2. Future studies focused on clinical outcomes, both MACEs and anginal relief, in patients with discordant FFR/NHPR results are needed to guide an optimal management strategy.
